# Laparoscopic excision for multiple intra-abdominal juvenile fibrosarcoma: A case report

**DOI:** 10.1016/j.ijscr.2021.106512

**Published:** 2021-10-18

**Authors:** Hanlim Choi, Jae-Woon Choi, Dong Hee Ryu, KangHe XU, Jin Young Lee, Chang Gok Woo

**Affiliations:** a Department of Surgery, Chungbuk National University Hospital, Cheongju, Republic of Korea; b Department of Surgery, Chungbuk National University College of Medicine, Cheongju, Republic of Korea; c Department of Trauma surgery, Chungbuk National University Hospital, Republic of Korea; d Department of Pathology, Chungbuk National University Hospital, Cheongju, Republic of Korea

**Keywords:** Fibrosarcoma, Juvenile, Intra-abdominal, Laparoscopy, Surgical excision

## Abstract

**Introduction and importance:**

Fibrosarcoma is a rare malignant tumor comprising spindle-shaped fibroblasts exhibiting variable collagen production. Adult-type fibrosarcoma (AFS) mainly occurs in people aged between 30 and 80 years, primarily in the deep soft tissues of the trunk, neck, and extremities, especially in areas surrounding bones. Juvenile fibrosarcoma(JFS) is a type of AFS that occurs in adolescents and rarely develops in the abdominal cavity.

**Case presentation:**

A 13-year-old girl presented with right upper quadrant pain for 5 days. Abdomen and pelvis computed tomography showed a 12 × 6-cm, ill-defined, lobulated, solid, cystic mass in the abdominal cavity. On laparoscopy, there were two masses in the abdominal cavity. One abutted the stomach and severely adhered to the gallbladder. The second mass was located between the transverse colon and duodenum, and it was surrounded by the omentum. The tissues surrounding the masses were finely dissected, and the two masses were excised completely. The patient was discharged without complications on post-operative day 7.

**Clinical discussion:**

JFS, AFS in adolescents, is a rare malignant tumor. And there have been no reported cases of multiple JFS in abdominal cavity. Surgical excision is the gold standard of treatment for localized AFS, and the laparoscopic approach for minimal tumor handling is beneficial.

**Conclusion:**

We describe a rare case of multiple intra-abdominal juvenile fibrosarcoma, managed through laparoscopic surgery.

## Highlights

• Fibrosarcoma is a rare malignant tumor composed of spindle-shaped fibroblasts with variable collagen production.
• Juvenile fibrosarcoma(JFS) is a type of AFS occurring in adolescents, and it rarely develops in the abdominal cavity.
• Curative surgical excision is the gold standard treatment for localized fibrosarcoma, and laparoscopic approach is benefitial.

## 1 Introduction

Fibrosarcoma is a rare malignant tumor composed of spindle-shaped fibroblasts with variable collagen production [1]. Fibrosarcomas can be classified into two types: the infantile or congenital type, and the adult type. Adult-type fibrosarcoma (AFS) mainly occurs in people aged between 30 and 80 years. It involves the deep soft tissues of the extremities, trunk, and neck [2]. Juvenile fibrosarcoma (JFS) is a type of AFS that occurs in adolescents, and rarely develops in the abdominal cavity. We describe a rare case of intra-abdominal juvenile fibrosarcoma that was managed through laparoscopic surgery. This work has been reported in line with the SCARE criteria [3].

## 2 Presentation of case

**Fig. 1:**
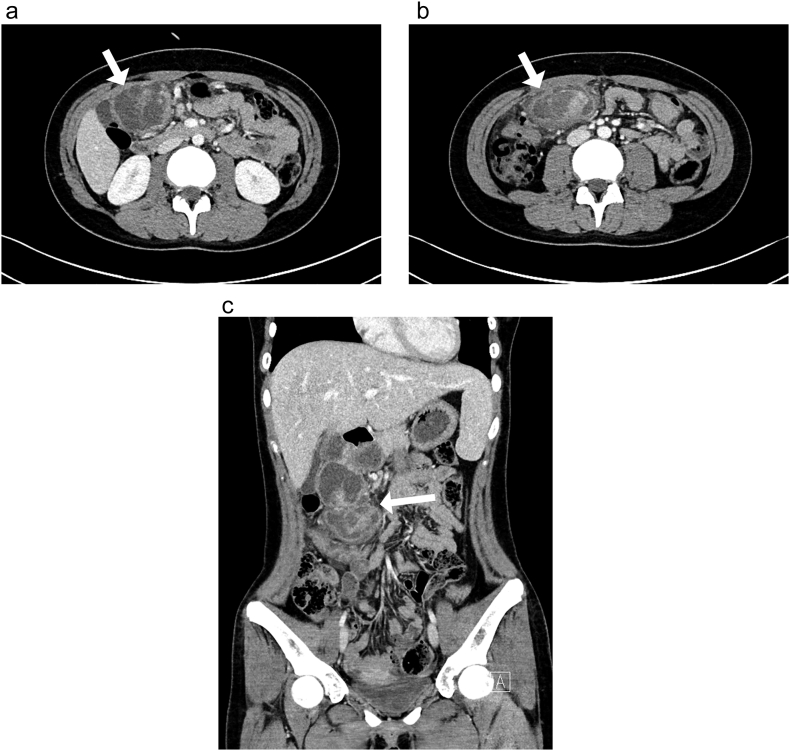
Contrast-enhanced abdomen-pelvis computed tomography. (A), (B) Axial view. (C) Coronal view. The mass on the right side of the greater omentum, abutting the left liver, gallbladder, and duodenal bulb (arrow).

**Fig. 2:**
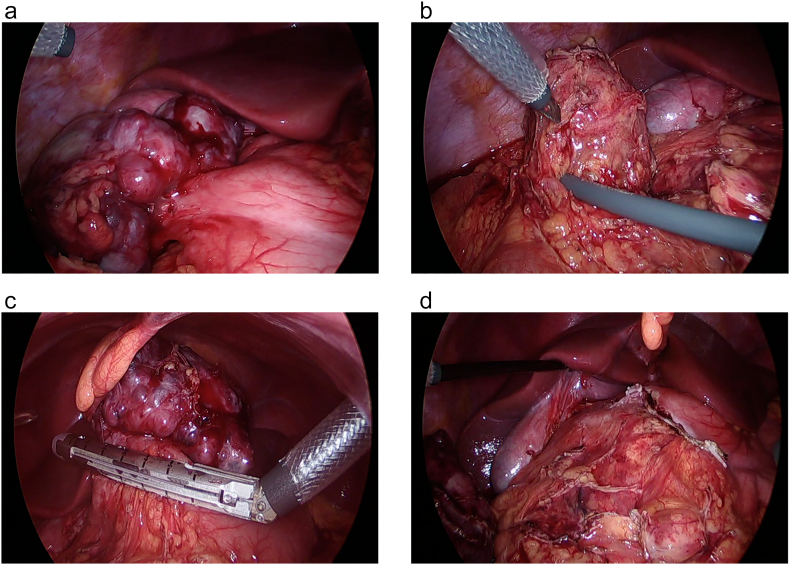
Laparoscopic view. (A) The first mass abutted the stomach and severely adhered to the gallbladder. (B) The second mass was located between the transverse colon and duodenum, surrounded by the omentum. (C), (D) After fine dissection around the masses, they were excised completely.

A 13-year-old girl presented with right upper quadrant pain for 5 days. On physical examination, there was no palpable abdominal mass, and her body mass index was 24 kg/m^2^. Her laboratory results were unremarkable. Contrast-enhanced abdomen and pelvis computed tomography showed a 12 × 6 cm, ill-defined, lobulated, solid, cystic mass in the abdominal cavity. The mass was located at the right side of the greater omentum, abutting the inferior aspect of the left liver, gallbladder, and the duodenal bulb and its second portion. The fat plane was preserved (Fig. 1A,B,C). In addition, the proximal part of the transverse colon exhibited segmental enhancing wall thickening with pericolic infiltration. An inflammatory myofibroblastic tumor was suspected, and laparoscopic exploration was performed. Three trocars were inserted: two of 12 mm trocars located on umbilicus and left upper quadrant area, one of 5 mm trocar located in right upper quadrant area. On laparoscopy, two masses were identified in the abdominal cavity. One abutted the stomach and severely adhered to the gallbladder (Fig. 2A). The second mass, surrounded by the omentum, was located between the transverse colon and duodenum (Fig. 2B). The tissue surrounding the masses were finely dissected, and the first mass abutting the stomach was excised using a linear stapler (Fig. 2C, D). The two resected masses were removed through an extended umbilical incision. On pathologic examination, both tumors were identified as AFS. The tumors comprised small-to-large spindled cells arranged in the fascicles or herringbone pattern. The tumor cells were membranous-positive for beta-catenin on immunohistochemistry. The patient was discharged without complications on post-operative day 7. The patient did not receive adjuvant therapy after surgery. She has been followed up for 2 years without any recurrence of tumor.

## 3 Discussion

This report highlights the case of a patient with multiple intra-abdominal AFS in adolescence, which is extremely rare disease.

Fibrosarcomas, malignant tumors originated from mesenchymal cell, are classified under fibroblastic/myofibroblastic sarcomas in the World Health Organization classification of soft tissue sarcomas [1]. There have been developments in diagnostic techniques, and histologically and genetically distinctive subtypes of fibrosarcoma have been identified. Fibrosarcomas are classified into infantile and adult types. Infantile fibrosarcomas rarely metastasizes, but AFS is highly malignant, locally aggressive, and frequently metastasizes [1], [2], [4]. AFS is composed of relatively monomorphic spindled cells exhibiting a mild to moderate degree of pleomorphism. On histology, they demonstrate the “herringbone” pattern with the absence of the ETV6-NTRK3 translocation. Immunohistochemistry can be applied to differentiate fibrosarcomas from other diseases by identifying the characteristic tumor markers [1], [2], [4].

According to recent reports, AFS accounts for 3.6% of sarcomas originating from soft tissues in adults [5]. In children and adolescents, rhabdomyosarcoma was the most common soft tissue sarcoma, and approximately 3% of childhood cancers were non-rhabdomyosarcoma cases. Among non-rhabdomyosarcomas, fibrosarcomas in adolescents were rare [6].

AFS mainly occurs in the deep soft tissues of the trunk, neck, and extremities, especially areas surrounding bones. And there have been no reports of multiple abdominal AFS lesions. Previous studies on AFS among pediatric patients did not identify the specific location of the tumor and the age of occurrence [6].

Since most AFS cases were diagnosed as high-grade lesions, which tend to behave aggressively, recur locally, and metastasize to the lymph and parenchyma, the prognosis is poor [2], [4], [7]. Curative surgical excision is the gold standard treatment for localized fibrosarcomas [4], [7]. And minimal tumor handling using the laparoscopic approach is beneficial. Surgical complete excision followed by adjuvant chemotherapy is not recommended for standard treatment because its effectiveness is unclear. However, adjuvant radiation therapy is recommended for high-grade and large tumors [4].

## 4 Conclusion

JFS, AFS in adolescents, is a rare malignant tumor. To the best of our knowledge, there have been no reported cases of multiple JFS in abdominal cavity. Surgical excision is the gold standard of treatment for localized AFS, and the laparoscopic approach for minimal tumor handling is beneficial.

## Funding

No funding.

## Ethical approval

This case report was approved by the institutional review board (IRB) of Chungbuk National University Hospital, South Korea (IRB No 2021–07-008).

## Patient consent

Written informed consent was obtained from a parent for the participant under 18 years old for publication of this article and any accompanying tables/images. A copy of the written consent is available for review by the Editor of this journal.

## CRediT authorship contribution statement

Conceptualization: Hanlim Choi, Dong Hee Ryu.

Data curation: Hanlim Choi, Chang Gok Woo, KangHe XU.

Investigation: Hanlim Choi, Jin Young Lee.

Supervision: Jae-Woon Choi, Dong Hee Ryu.

Writing – original draft: Hanlim Choi.

Writing – review & editing: Hanlim Choi, Jin Young Lee, Dong Hee Ryu.

## Registration of research studies

Not applicable.

## Guarantor

Dong Hee Ryu.

## Provenance and peer review

Not commissioned, externally peer-reviewed.

## Declaration of competing interest

The authors declare that they have no competing interests.
